# Exploring the role of RRM domains and conserved aromatic residues in RGG motif  of eIF4G-binding translation repressor protein Sbp1

**DOI:** 10.12688/wellcomeopenres.14709.2

**Published:** 2020-02-06

**Authors:** Nupur Bhatter, Rajan Iyyappan, Purusharth I Rajyaguru

**Affiliations:** 1 Indian Institute of Science, Bangalore, India; 2 IAPG, Prague, Czech Republic

**Keywords:** mRNA fate decisions, Translation control, RGG-motif, Sbp1, eIF4G, Translation repression

## Abstract

**Background: **Mechanisms of mRNA fate decisions play an important role in determining if a given mRNA will be translated, stored or degraded upon arrival to cytoplasm. Sbp1 is an important RGG-motif containing protein that is implicated in affecting mRNA decapping and translation. Sbp1 represses translation by binding eIF4G1 through its RGG-motif and activates decapping when overexpressed. In this report we have assessed the genetic interaction of Sbp1 with  decapping activators such as Dhh1, Pat1 and Scd6.  We have further analyzed the importance of different domains and specific conserved residues of Sbp1 in translation repression activity. **Method: **Sequence alignment was performed to identify conserved aromatic residues to be mutated. Using site-directed mutagenesis several point mutations and domain deletions was created in Sbp1 expressed under a galactose-inducible promoter. The mutants were tested for their ability to cause growth defect upon over-expression. The ability of Sbp1 to affect over expression mediated growth defect of other decapping activators was tested using growth assay. Live cell imaging was done to study localization of Sbp1 and its RRM-deletion mutants to RNA granules upon glucose starvation.

**Results: **Mutation of several aromatic residues in the RGG-motif and that of the phosphorylation sites in the RRM domain of Sbp1 did not affect the growth defect phenotype. Deletion of another eIF4G1-binding RGG-motif protein Scd6 does not affect the ability of Sbp1 to cause growth defect. Moreover, absence of Sbp1 did not affect the growth defect phenotypes observed upon overexpression of decapping activators Dhh1 and Pat1. Strikingly deletion of both the RRM domains (RRM1 and RRM2) and not the RNP motifs within them compromised the growth defect phenotype. Sbp1 mutant lacking both RRM1 and RRM2 was highly defective in localizing to RNA granules.

**Conclusion: **This study identifies an important role of RRM domains independent of RNP motif in Sbp1 repression activity.

## Introduction

Regulation of mRNA stability and translation plays a key role in cellular processes. RNA binding proteins orchestrate such regulatory processes. Translation repressors are an important class of RNA binding proteins that regulates mRNA fate in the cytoplasm. RGG-motif containing proteins have recently emerged as an exciting class of RNA-binding proteins. A subset of RGG-motif proteins has recently been reported to repress translation by binding eIF4G1 ( Rajyaguru *et al.*, 2012; Rajyaguru & Parker, 2012).

**Figure 1.**
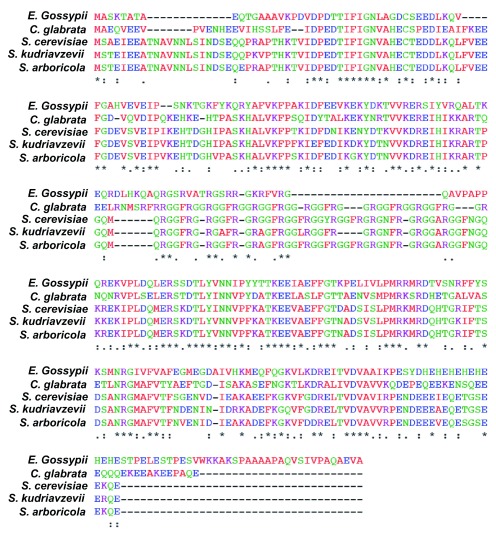
Alignment of Sbp1 amino acid sequence from different genuses of family Saccharomycetaceae using T-COFFEE multiple sequence alignment tool. Multiple sequence alignment of Sbp1 reveals conservation of aromatic residue in the RGG motif and RNP sequence in the RRM domain. Blue indicate negatively charged amino acids, purple indicate positively charged amino acids, red indicate hydrophobic amino acids and green indicate polar uncharged amino acid.

**Figure 2.**
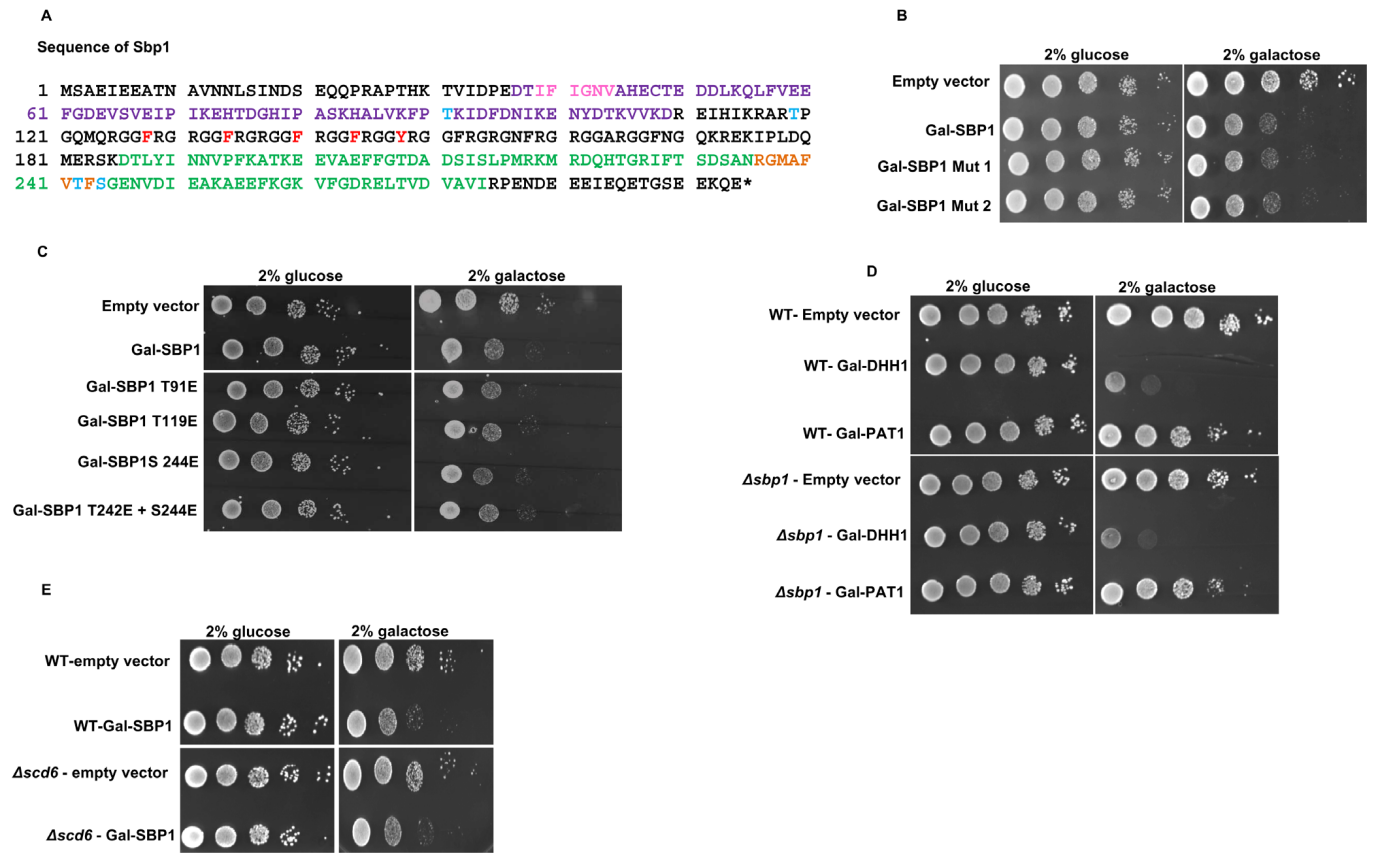
Effect of aromatic to alanine and phosphomimetic mutations in Sbp1 overexpression mediated growth defect and genetic interaction between Sbp1 and decapping activators such as Scd6, Dhh1 and Pat1. **A**. Amino acid sequence of Sbp1 showing different domains and motifs. Specific threonine and serine residues that were mutated to glutamic acid are marked blue. Purple region marks RRM1 sequence, pink denotes RNP2 sequence, green region denotes RRM2 sequence and orange denotes RNP1 sequence. Aromatic amino acid in RGG motif are marked red. Mut 1 has F128, F133 and F139 (marked red) mutated to alanine. Mut 2 has F143, Y147 and F151 (maroon) mutated to alanine along with the mutations present in Mut.1. **B**. Growth assay showing role of aromatic residues in the RGG motif on over expression mediated growth defect of Sbp1. **C**. Growth assay images showing effect of constitutive phosphorylation on Sbp1 over expression mediated growth defect. **D**. Growth assay showing effect of Sbp1 deletion on growth defect phenotype of translation repressors Pat1 and Dhh1. **E**. Growth assay showing effect of Scd6 deletion on Sbp1 over–expression mediated growth defect. Top and bottom panels respectively in **D** and **E** are cropped from the same assay plate.

Sbp1 was identified as a single stranded nucleic acid binding protein ( Jong & Campbell, 1986). It can act as a decapping activator and translation repressor ( Segal *et al.*, 2006). Consistent with its role in translation and mRNA decay it can bind mRNA and localizes to RNA granules such as P-bodies and stress granules in response to glucose deprivation ( Mitchell *et al.*, 2013). Sbp1 is a modular protein ( Figure 2A) with two RNA Recognition Motifs (RRMs) sandwiching a central RGG-motif ( Rajyaguru *et al.*, 2012). The RGG-motif is important for the translation repression activity of Sbp1. Interestingly the RGG-motif is interjected with aromatic residues (specifically phenylalanine and ‘FRG’ repeats) [ Figure 1 and Figure 2A, the relevance of which is unclear.

RGG-motif of Sbp1 targets eIF4G1 to repress translation. During translation initiation, eIF4G plays an important role as a scaffolding initiation factor that recruits other initiation factors such as eIF4E, eIF4A and Pab1 ( Merrick, 2015) to orchestrate formation of the cap-binding complex. Identification of several RGG-motif proteins that bind eIF4G to repress translation indicated that the role of these proteins in translation initiation could be mRNP-specific. RNA-binding domains fused to the RGG-motif could orchestrate such specificity. The RRM domains could be performing similar function for Sbp1 however their contribution to Sbp1 repression activity has not been tested.

Interestingly, Sbp1 is phosphorylated and arginine methylated ( Albuquerque *et al.*, 2008; Frankel & Clarke, 1999; Swaney *et al.*, 2013). The significance of arginine methylation has recently been reported in promoting translation repression activity of Sbp1 ( Bhatter *et al.*, 2019). However the relevance of Sbp1 phosphorylation remains unclear. Interestingly the RGG-motif of Sbp1 is interspersed with aromatic amino acid residues that are conserved ( Figure 1). A similar pattern is observed in the RGG-motif of Scd6 ( Roy & Rajyaguru, 2018). The importance of aromatic residues in RGG-motif has not been explored.

In this work, we have attempted to understand the following four aspects of Sbp1 function, a) the role of aromatic residues in the RGG-motif, b) the role of phosphorylation site, c) genetic interactions of Sbp1 with decapping activators such as Scd6, Pat1 and Dhh1 and d) the contribution of two RRM domains towards Sbp1 function.

## Results

### Mutation in aromatic residues of RGG motif does not affect growth defect upon Sbp1 over expression

Sbp1 contains 8 aromatic residues (7 phenylalanine and 1 tyrosine) in the RGG-domain (125–165). Out of these, 6 phenylalanine occur as ‘FRG’ repeats interspersed with ‘RGG’ repeats. Alignment of the Sbp1 protein sequence revealed that the ‘FRG’ repeats are fairly conserved in other *Saccharomyces* species as well as in *Candida glabrata* ( Figure 1). Abundance of aromatic residues in RGG-motif is also observed with another RGG-motif protein Scd6. Aromatic amino acids have been fairly well characterized in RNA binding proteins and reported to contribute to RNA-binding through base stacking interactions ( Moras & Poterszman, 1995; Rahman *et al.*, 2015). Specifically aromatic residues surrounded by charged residues ( Figure 2A) have been implicated in RNA binding for example in the case of the RNP1 and RNP2 sequence motifs present in RNA Recognition Motifs (RRMs) ( Maris *et al.*, 2005). This led us to hypothesize that the conserved aromatic residues in the RGG-motif of Sbp1 could contribute to the repression activity of Sbp1 presumably through binding RNA. We decided to test the importance of these residues using a simple overexpression growth assay. Sbp1 overexpression leads to growth defect phenotype and mutants of Sbp1 defective in translation repression activity indicate compromised growth defect phenotype ( Bhatter *et al.*, 2019). We mutated phenylalanine and tyrosine residues to alanine. We observe that mutating up to 5 phenylalanine and 1 tyrosine residue (Mut2) to alanine did not affect the ability of Sbp1 to cause a growth defect upon overexpression ( Figure 2B). Based on the growth assay we conclude that mutated aromatic residues in RGG-motif perhaps do not play a very important role in Sbp1 over expression mediated growth defect.

### Phospho-mimetic mutants of Sbp1 do not alter growth defect phenotype upon overexpression

Sbp1 gets phosphorylated at T91, T119, T242 and S244 ( Figure 2A) ( Albuquerque *et al.*, 2008; Holt *et al.*, 2009; Swaney *et al.*, 2013). Phosphorylation is a common posttranslational modification that regulates protein function by altering protein-protein and/or protein-nucleic acid interaction. To test if phosphorylation of reported threonine and serine could alter Sbp1 repression activity we created four different phospho-mimetic mutants (T91E, T119E, T242E and S244E). Amongst the known phosphorylation residues, T91 and T119 has been reported to get phosphorylated upon MMS treatment which causes DNA damage ( Albuquerque *et al.*, 2008). We hypothesized that if phosphorylation were required to activate the repression activity of Sbp1 then phospho-mimetic mutants could lead to a stronger growth defect phenotype. We observed that none of the mutations affected the ability of Sbp1 to cause growth defects upon overexpression ( Figure 2C). This indicated that phosphorylation of the specific residues that were tested did not affect the repression activity of Sbp1 based on the growth assay.

### Scd6 does not affect the ability of Sbp1 to cause growth defect

Scd6, like Sbp1 is an RGG-motif containing translation repressor protein that binds eIF4G1 to repress translation ( Roy & Rajyaguru, 2018). The significance of multiple RGG-motif containing translation repressor proteins binding eIF4G is unclear. It is possible that one RGG-motif containing repressor could affect the activity of another repressor. To address this, we decided to test the dependency of Sbp1 on Scd6 in causing growth defect upon over expression. We observed that absence of Scd6 did not alter the growth defect phenotype observed upon Sbp1 overexpression ( Figure 2E). Based on these observations we conclude that Scd6 does alter the ability of Sbp1 to cause growth defect. The reported role of Sbp1as a decapping activator ( Segal *et al.*, 2006) prompted us to test if Sbp1 could modulate the repression activity of other decapping activators and translation repressors such as Dhh1 and Pat1 ( Coller & Parker, 2005; Pilkington & Parker, 2008).

We overexpressed Dhh1 and Pat1 in wild type and *Δsbp1* background. Absence of Sbp1 did not affect the growth defect observed upon Dhh1 and Pat1 overexpression ( Figure 2D). This result indicates that Sbp1 does not alter overexpression growth defect of Dhh1 and Pat1.

### Deletion of RRM domains of Sbp1 compromises overexpression mediated growth defect

**Figure 3.**
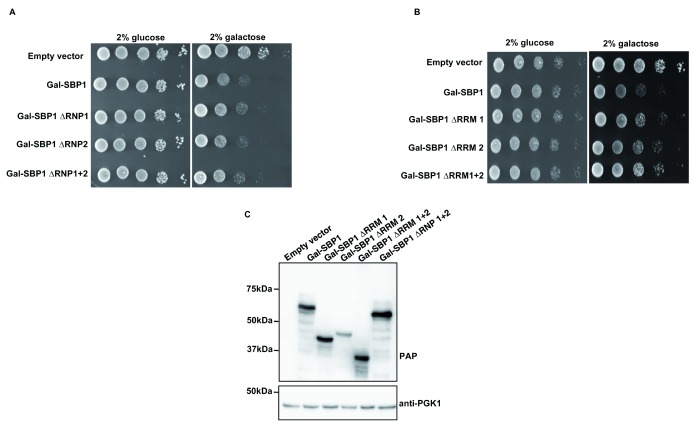
Effect of RRM domain and RNP-motif sequence deletion on Sbp1 over expression mediated growth defect. **A**. Growth assay of Sbp1 RNP1, RNP2 and RNP 1+2 sequence deletion mutants (as indicated in Figure 2A). **B**. Growth assay with ∆RRM1, ∆RRM2 and ∆RRM 1+2 mutants of Sbp1. Images in both panels have been cropped from the same growth assay plate. **C**. Western blot using PAP antibody (Sigma) showing protein level of WT and indicated mutants of Sbp1. PAP will recognize the ZZ tag present in wild-type as well as Sbp1 mutants at the C-terminus. PGK1 served as loading control. Blot was first probed with PAP and then stripped followed by probing with anti-PGK1 antibody (Abcam).

RRM domains are highly conserved domains involved in binding both RNA and protein ( Maris *et al.*, 2005). Two RRM domains that contain conserved RNP-motif sequence flank Sbp1 RGG motif. The RNP motifs contribute to the RNA binding activity in RRM domains. To test the role of RNP motifs we created deletions of RNP1, RNP2 and RNP1+RNP2. None of these mutations strongly affected the ability of Sbp1 to cause growth defect upon overexpression ( Figure 3A). We next created deletion of RRM1, RRM2 and RRM1+2. Deletion of both the RRM domains led to partially compromised growth defect phenotype ( Figure 3B) whereas the deletion of individual RRM1 or RRM2 domains compromised the growth defect phenotype to a lesser extent than RRM1+2 ( Figure 3B). Western blot analysis indicates that RRM1+2 mutant is expressed in manner comparable to wild type ( Figure 3C). Based on these results we conclude that the RRM domains are important for causing growth phenotype. Since RNP1+2 deletion did not affect the growth defect phenotype, we interpret this result to suggest that the growth defect is independent of the RNP motifs.

### Deletion of both the RRM domains impair the ability of Sbp1 to localize to granules upon glucose starvation

**Figure 4.**
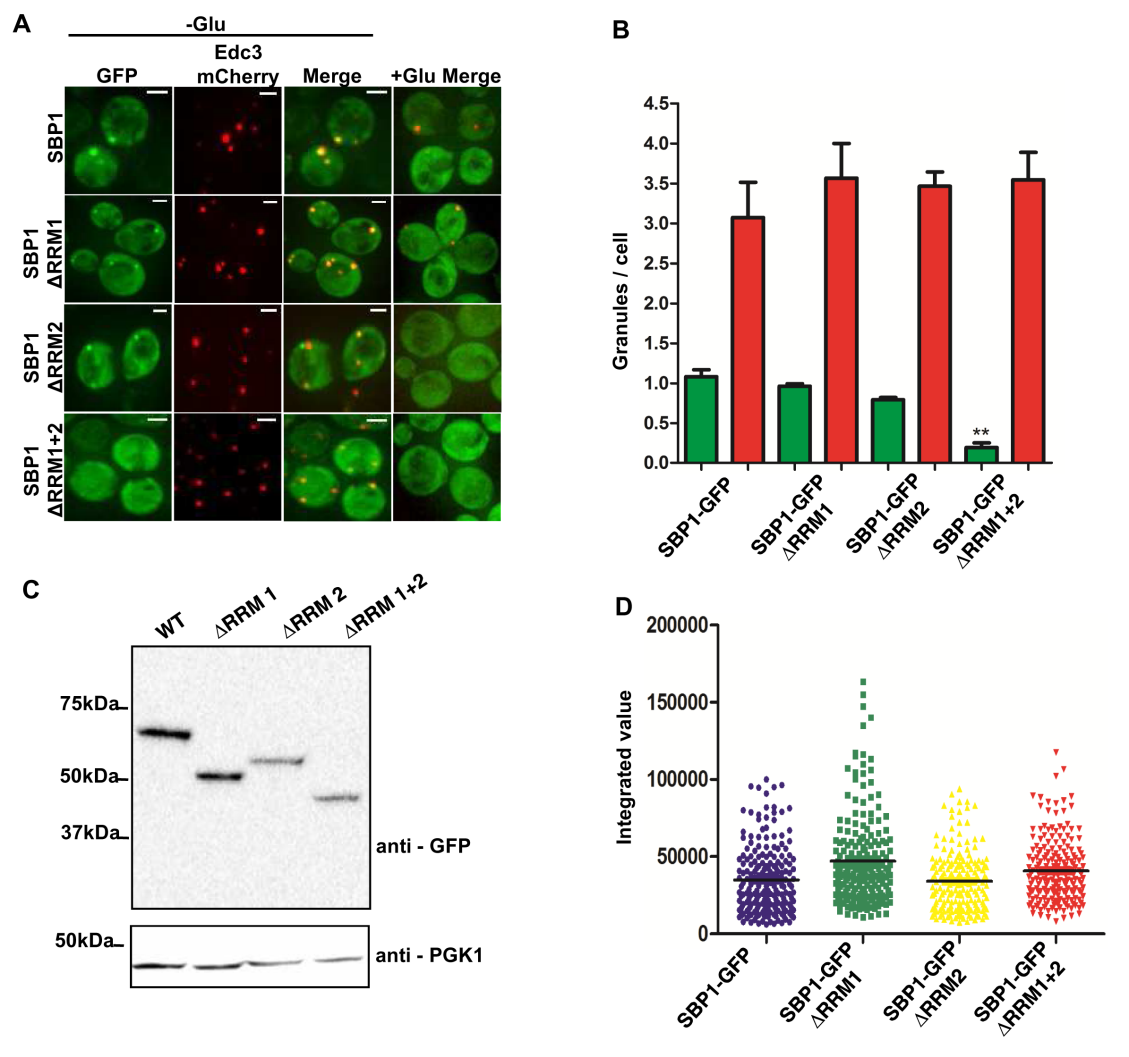
Effect of double deletion of RRM domains of Sbp1 on its ability to localize to RNA granules upon stress. **A**. Live cell imaging of WT and RRM domain deletion mutants of Sbp1 after 10 min of glucose starvation. Sbp1 was tagged to GFP and Edc3 was tagged to mCherry. Normally grown and glucose deprived cells were pelleted together at 14,100rpm for 12 s. For all experiments, glucose-starved cells were imaged first followed by cells grown with glucose. Scale denotes 2µm. **B**. Graph plotted for Sbp1-GFP and Edc3-mcherry granules per cell for n=3. p value for Sbp1-GFP wt and ∆RRM1+2 mutant is 0.0071. Asterisks denote statistical significance of the data plotted (** = p value> 0.01 and * = p value > 0.05). Red bar denotes Edc3-mCherry granule number and green denotes GFP granule number. **C**. Protein levels of WT Sbp1-GFP and its mutants treated with sodium azide. Blot was first probed with anti-GFP antibody followed by stripping and probing with anti-PGK antibody. **D**. Intensity of GFP in cells expressing GFP tagged Sbp1 wt and mutants, quantitated from images of glu starved cells (n=150).

Sbp1 has been reported to localize to RNA granules upon glucose starvation which colocalizes with Edc3mcherry and Pub1mcherry ( Mitchell *et al.*, 2013; Segal *et al.*, 2006). The compromised ability of RRM1+2 mutants in causing a growth defect could be due to its defective localization to RNA granules. We tested if Sbp1 RRM1+2 mutant localized to RNA granules in response to glucose starvation by using a construct where Sbp1 is under its own promoter (500 base-pairs upstream of start codon) and tagged to GFP (followed by ADH terminator sequence) at its C-terminus. We observed that Sbp1 localized to RNA granules upon glucose starvation stress as reported earlier but RRM 1+2 deletion mutant was impaired in its ability to localize to RNA granules ( Figure 4A & B). Mutants with individual RRM domain deletion did not show a significant defect in localization to RNA granules upon glucose starvation compared to wild-type ( Figure 4A & B). Localization of Edc3-mcherry in the cells expressing wild type or mutant Sbp1-GFP to granules was comparable suggesting that the inability of RRM 1+2 mutant to localize to RNA granules was not due to inadequate stress. Defective localization to RNA granules was not due to decreased protein expression since the RRM1+2 deletion mutant was expressed in manner comparable to the wild type protein ( Figure 4C and D).

This result indicates that RRM domains are required for the localization of Sbp1 to RNA granules upon glucose starvation stress.

## Discussion

In this work, we provide evidence that a) aromatic (phenylalanine and tyrosine) residues interspersed in the RGG-motif and phosphorylation sites in the RRM domain of Sbp1 do not contribute to overexpression growth defect ( Figure 2A-C) b) RGG-motif protein Scd6 does not affect the ability of Sbp1 to reduce growth upon over expression ( Figure 2E) c) Sbp1 does not affect the ability of Dhh1 and Pat1 to cause growth defect ( Figure 2D) d) Deletion of RRM domains compromises overexpression mediated growth defect ( Figure 3A & B) e) RRM domains of Sbp1 are required for localization to RNA granules upon glucose starvation ( Figure 4A & B).

Aromatic residues have been implicated in RNA-binding through base stacking interactions. Sbp1 binds a subset of mRNA in yeast ( Mitchell *et al.*, 2013). Sbp1 has two RRM domains, which are likely to be involved in RNA binding. RGG-motif have also been reported to bind RNA, specifically G-rich structures such as G-quadruplex ( Fay *et al.*, 2017). Whether Sbp1 binds RNA with G-quadruplex structure is not known however we decided to test the role of RGG-motif phenylalanine and tyrosine in Sbp1 repression activity through growth assays. Sbp1 mutant with 6 aromatic residues converted to alanine upon overexpression leads to a growth defect comparable to wild type Sbp1 ( Figure 2B) indicating that the phenylalanine residues do not contribute to the growth defect.

The phosphorylation sites in Sbp1 are not in the RGG-motif, which is important for Sbp1 repression activity. The significance of Sbp1 phosphorylation is not known. We tested the contribution of phosphorylation sites in Sbp1 and observed that three single, and one double phospho-mimetic mutant did not change the growth defect phenotype caused by over expression of Sbp1 ( Figure 2C). It is possible that phospho-mimetic mutation of all sites simultaneously or creating phospho-dead mutants could provide further insight into the role of phosphorylation in Sbp1 function.

Even though both Scd6 and Sbp1 bind eIF4G to repress translation, Scd6 does not affect the ability of Sbp1 to repress translation, as deletion of Scd6 did not alter growth defect upon Sbp1 overexpression ( Figure 2E). This result points to the idea that despite targeting the same initiation factor both Scd6 and Sbp1 might have non-overlapping mRNA targets. Scd6 contains Lsm and FDF domains as RNA-binding domains, whereas RRMs are the RNA-binding domains of Sbp1. Comparing mRNA targets of Sbp1 and Scd6 would be an important future direction to understand the details of their repression activity.

It was recently demonstrated that Sbp1, Dhh1 and Pat1 bind to common mRNA subsets suggesting a cumulative role of these factors in affecting translation and/or stability of target mRNAs ( Mitchell *et al.*, 2013). We observe that absence of Sbp1 did not affect overexpression growth defect phenotype of Dhh1 and Pat1 ( Figure 2D). It must be noted that Pat1 overexpression has a very weak growth defect phenotype.

RNA recognition motif (RRM) is well known RNA binding domain present in proteins that are involved in RNA metabolism. This domain is often fused to RGG motif in proteins such as FUS, Nucleolin and TDP43 in mammals. Two RRM domains flank Sbp1 RGG motif and deletion of both the RRM domains of Sbp1 led to partial rescue of growth defect ( Figure 3B) indicating that RRM domains along with the previously reported RGG-motif are important for growth defect phenotype and likely Sbp1 repression activity. The microscopy data provides further clear indication about the role of RRM domain in Sbp1 repression activity. Deletion of both the RRM domains renders the localization of Sbp1 to RNA granules defective upon glucose starvation ( Figure 4A & B). Inability of the RRM deletion mutant to localize to RNA granules could be due to defective interaction with either mRNAs or a granule-resident protein that guides the localization of Sbp1 to granules or both. Surprisingly the deletion of consensus RNP motif sequences (RNP 1+2) did not affect the growth defect phenotype ( Figure 3A). This indicates that the site(s) required for Sbp1 interaction with mRNA and/or protein for translation repression function is not in the RNP motifs. Identifying the mRNAs and/or proteins bound by the Sbp1 RRM domains will be an important future direction.

Overall our growth-assay and live cell imaging based study provides insights into the role of RRM domains in Sbp1 repression activity. It identifies a positive role of RRM domains in Sbp1 repression activity paving the way for addressing the mechanistic basis of the role of RRM domains in Sbp1 function.

## Methods

### Yeast strains and plasmids

All strains, plasmids and oligos used in this study are listed in Supplementary Table 1, 2 and 3 respectively. Please see ‘Data availability’ section below for more details regarding these tables. Yeast strains used in this study are BY4741 (wild type), *Δsbp1*(YSC1053, Dharmacon) and *Δscd6*. Strains were grown on synthetic medium (SC) supplemented without uracil and 2% glucose (51758, Sisco Research Laboratories) or galactose (G0750, Sigma Aldrich). All strains were grown at 30°C. pPIR6 is BG1805 empty vector, a kind gift from Roy Parker lab ( Nissan *et al.*, 2010).

### Site-directed mutagenesis

For creating point mutations in construct expressing galactose-inducible Sbp1, primers were designed using Quick change primer design tool from Agilent. The oligos were procured from Bioserve Biotechnologies. Phusion taq polymerase (FNZ520S, Thermo Fisher) was used for PCR. 4 cycles of PCR was done with forward and reverse primers (Figure S3)(Bioserve Biotechnologies) in different vial along with PCR reaction mixture using thermal cycler (6331000017, Eppendorf). The conditions of PCR were as follows: initial denaturation at 98°C for 10 minutes followed by cycles of denaturation at 98°C for 30 seconds, annealing at 55°C for 30 seconds, extension at 72°C for 4 minutes 30 seconds and final extension at 72°C for 10 minutes. This step allows amplification of single strand of the plasmid with mutation in desirable position as present in the primer. Before starting the next 21 PCR cycles (cycling conditions used were same as above), the contents of the two tubes were mixed and put in same vial. After PCR, the reaction mixture was subjected to treatment with Dpn1 (ER1701, Thermo Scientific) restriction enzyme (before adding the enzyme, 1/5 ^th^ volume of the reaction was taken out to be used as control). Both treated and untreated PCR reaction mixture were then transformed in *E.coli* XL1BLUE strain(a kind gift from Parker lab) and selected in Luria Bertini (L.B) agar plates(‘Molecular Cloning: A Laboratory Protocol, CSHL press)supplemented with 100ug/ul of ampicillin (61314, Sisco Research Laboratories). Components for media were procured from Himedia labs (Tryptone–RM014, Peptone-RM001, Yeast extract-RM027, Agar-RM301 and GRM026, Sodium Chloride- 33205). Colonies obtained by transforming Dpn1-treated PCR-mix were screened for mutation and confirmed by colony PCR (wherever applicable) and Sanger sequencing (Medauxin, Bangalore).

### Growth assays

All strains were patched on synthetic medium without uracil and allowed to grow overnight. Next day cells from patches were re-suspended and Optical density of culture was measured at 600 nm wavelength using water as blank with the help of spectrophotometer (6133000907, Eppendorf). The following dilutions were prepared 10, 1, 0.1, 0.01 and 0.001 in 96-well plates. In all the growth assays 5 µl of diluted culture was spotted on both SD-URA plates with 2% glucose and 2% galactose. Glucose and galactose plates were imaged at 36–48 h and 60–72 h timeframe respectively using gel documentation system (Image Quanta LAS 4000, GE Healthcare). The settings of camera for imaging were tray position 2, precision setting, 1/30 seconds and brightness at 6.

### Live cell imaging

For glucose starvation stress with SBP1-GFP construct, yeast cultures were grown to OD _600_ of 0.5–0.6 in SD-Leu-ura + 2% glucose media at 30°C. Glucose starvation was done as described previously ( Bhatter *et al.*, 2019). Briefly, after reaching desired O.D, cells were split into two equal volume followed by pelleting at 4200rpm for 10s at room temperature in eppendorf centrifuge. This was followed by washing cells with respective media (-glu pellet with SD URA- without glucose and +glu pellet with SD URA- media with glucose media). Final resuspended cells were allowed to grow for 10min in shaker incubator. This was followed by pelleting cells at 14200 rpm for 12s and spotting them on coverslip to observe under microscope at room temperature.All images were acquired using Deltavision Elite microscope (GE Healthcare) system running softWoRx 3.5.1 software (Applied Precision, LLC), using an Olympus 100×, oil-immersion 1.4 NA objective. Exposure time and transmittance for Green Fluorescent Protein (GFP) channel was 0.2 seconds and 32% respectively. Exposure time and transmittance for mCherry channel were 0.3 seconds and 32% respectively. Images were collected as 512 × 512 pixel files with a CoolSnapHQ camera (Photometrics) using 1 × 1 binning for yeast. All yeast images were deconvolved using standard softWoRx deconvolution algorithms. ImageJ was used to adjust all images to equal contrast ranges according to the experiment conducted or protein examined. For Sbp1-GFP experiment on an average, minimum of 100 cells was counted per experiment. Data from three independent experiments was used for quantitation and statistical significance was calculated using two-tailed paired *t*-test. Quantitation of intensity for glucose starved GFP cells were done as described previously ( Parbin *et al.*, 2020).

### Western blotting

To look at the protein level of wild type and mutants of Sbp1 in BG1805 construct, cells were first grown in SD ura-minimal media with glucose till 0.45–0.5 OD600 this was followed by pelleting and growing in SD ura- 2% galactose overnight. Cells were broken open using acid wash glass beads and 20microgram of total protein was loaded in 8% SDS polyacrylamide gel. The gel was transferred onto a nitrocellulose membrane using Bio-Rad wet transfer apparatus. Post transfer, the membrane was stained with Ponceau S to know the total protein present in each lane. The blot was washed and blocked using skimmed milk. PAP (1:5000, Sigma Aldrich cat# P1291) was used to detect over expressed Sbp1 and mutant proteins. Sbp1-GFP and its mutants protein level was looked at the same way as gal inducible Sbp1 except the use of anti-GFP anti body (1: 1000, BioLegend cat# 902602). For loading control, blot was stripped and put in anti - PGK1 antibody (1:1000, Abcam cat# AB113687).

## Data availability

### Underlying data

The data underlying this study is available from Open Science Framework. Dataset 1: Characterizing mutations in and genetic interactions of RGG-motif translation repressor Sbp1 https://doi.org/10.17605/OSF.IO/ZVRH7 ( Rajyaguru, 2018).

### Extended data

The supplementary tables (see Methods above) listed below have been uploaded on Figshare

DOI 10.6084/m9.figshare.11733786

Supplementary Table 1: List of strains.

Supplementary Table 2: List of plasmids.

Supplementary Table 3: List of oligonucleotides.
