# Supplementary material for: Exploring the role of RRM domains and conserved aromatic residues in RGG motif  of eIF4G-binding translation repressor protein Sbp1
Source: Wellcome Open Res. 2021 Sep 17;3:102. Originally published 2018 Aug 22. [Version 3] doi: 10.12688/wellcomeopenres.14709.3 (PMC7059846; doi:10.12688/wellcomeopenres.14709.3)
Supplement: Peer review history

# Peer review: Reviewer response for version 3

**DOI:** 10.21956/wellcomeopenres.18850.r45934

**Published:** 2021-09-21

**Authors:** Jeffrey Wilusz

**Affiliations:** 1 Department of Microbiology, Immunology and Pathology, Colorado State University, Fort Collins, CO, USA

I find the revised version to be improved and largely responsive to the points that I raised in the initial round of critiques. I have no further comments to make.

Is the work clearly and accurately presented and does it cite the current literature?

Yes

If applicable, is the statistical analysis and its interpretation appropriate?

Yes

Are all the source data underlying the results available to ensure full reproducibility?

No source data required

Is the study design appropriate and is the work technically sound?

Partly

Are the conclusions drawn adequately supported by the results?

Partly

Are sufficient details of methods and analysis provided to allow replication by others?

Yes

Reviewer Expertise:

mRNA stability; RNA virus host interactions; RNA biology.

I confirm that I have read this submission and believe that I have an appropriate level of expertise to confirm that it is of an acceptable scientific standard.

# Peer review: Reviewer response for version 2

**DOI:** 10.21956/wellcomeopenres.17163.r37997

**Published:** 2020-03-04

**Authors:** Sarah F. Mitchell

**Affiliations:** 1 Department of Chemistry and Biochemistry, Loyola Marymount University, Los Angeles, CA, USA

This work investigates the growth defect caused by over expression of the translational repressor Sbp1. The roles of the RRM domains, individual amino acids in the RGG domain, and phosphorylated amino acids are investigated. Though most of the results are negative, deletion of both RRM domains leads to a subtle reduction in the over-expression growth defect and a reduction in the presence of Sbp1 in RNA granules. These findings are minor, but will be of interest to researchers in the field. Some of the findings could be more strongly supported by a clearer presentation of several figures. I have several specific, predominantly minor, suggestions listed below.

1. In Figure 1, it would be helpful to the reader if the location of the RRM and RGG domains could be indicated. 
2. In Figure 2A, the numbering in the sequence is not consistent with the numbering of the mutants described in the figure legend. The numbering for F133, F139, F143, Y147 and F151 appears to be shifted by one amino acid such that in the sequence the numbers appear to be higher (F134, F140, etc.). In addition, F151 is not colored as indicated in the legend. 
3. In Figure 3B, the reduced growth defect is subtle. As mentioned by other reviewers, a growth curve would strengthen the evidence for this change. Another way to make the difference appear more convincing may be to present images that are more consistent. In this figure, the background for the 2% galactose is much darker than other figures in the manuscript. This makes a direct comparison more difficult. 
4. Figure 3C shows a reduced amount of Gal-SBP1deltaRRM2 relative to other constructs. This does not alter the primary conclusion that deletion of both RRMs reduces the growth defect, but may alter the more minor conclusion that there is a lesser reduction in the growth defect for the individual RRM deletions. It should be mentioned in the manuscript. 
5. In Figure 4A, the Edc3-mCherry panels do not align well with the GFP panels. This makes the results more difficult to interpret. 
6. The authors write that the western blot in Figure 4C shows comparable expression of WT and deltaRRM1+2 proteins. As the control bands (Pgk1) appear different in these two samples, quantification of this blot would make this statement more convincing.

Is the work clearly and accurately presented and does it cite the current literature?

Partly

If applicable, is the statistical analysis and its interpretation appropriate?

Not applicable

Are all the source data underlying the results available to ensure full reproducibility?

Yes

Is the study design appropriate and is the work technically sound?

Yes

Are the conclusions drawn adequately supported by the results?

Yes

Are sufficient details of methods and analysis provided to allow replication by others?

Yes

Reviewer Expertise:

mRNA-protein interactions, translation, post-transcriptional regulation of gene expression

I confirm that I have read this submission and believe that I have an appropriate level of expertise to confirm that it is of an acceptable scientific standard.

# Response

**Published:** 2021-08-03

**Authors:** Purusharth Rajyaguru

**Affiliations:** Indian Institute of Science, India

**Sarah F. Mitchell**

**Department of Chemistry and Biochemistry, Loyola Marymount University, Los Angeles, CA, USA**

 This work investigates the growth defect caused by over expression of the translational repressor Sbp1. The roles of the RRM domains, individual amino acids in the RGG domain, and phosphorylated amino acids are investigated. Though most of the results are negative, deletion of both RRM domains leads to a subtle reduction in the over-expression growth defect and a reduction in the presence of Sbp1 in RNA granules. These findings are minor, but will be of interest to researchers in the field. Some of the findings could be more strongly supported by a clearer presentation of several figures. I have several specific, predominantly minor, suggestions listed below.

**Author’s comment:** The authors thank the reviewer for all the comments.

 1. In Figure 1, it would be helpful to the reader if the location of the RRM and RGG domains could be indicated.

**Author’s comment:** The details of RRM and RGG domain with amino acid number and location has been indicated in figure 2A cartoon. Further explanations are provided in the figure legends.

 2. In Figure 2A, the numbering in the sequence is not consistent with the numbering of the mutants described in the figure legend. The numbering for F133, F139, F143, Y147 and F151 appears to be shifted by one amino acid such that in the sequence the numbers appear to be higher (F134, F140, etc.). In addition, F151 is not colored as indicated in the legend.

**Author’s comment:** We have incorporated all the changes in Figure 2. The correct amino acid mutation for Mut1 is F128, F134 and F140 (marked red) which were mutated to alanine. Mut 2 has F144, Y148 and F152 in addition to mutations present in Mut1. All the corrected amino acid number are mentioned in the legend.

 3. In Figure 3B, the reduced growth defect is subtle. As mentioned by other reviewers, a growth curve would strengthen the evidence for this change. Another way to make the difference appear more convincing may be to present images that are more consistent. In this figure, the background for the 2% galactose is much darker than other figures in the manuscript. This makes a direct comparison more difficult.

**Author’s comment:** We have repeated growth assay and replaced the older image with new one in Figure 3B. ∆RRM1+2 mutant of SBP1 has consistently shown partial rescue form over-expression mediated growth defect in spotting assays.

 4. Figure 3C shows a reduced amount of Gal-SBP1deltaRRM2 relative to other constructs. This does not alter the primary conclusion that deletion of both RRMs reduces the growth defect, but may alter the more minor conclusion that there is a lesser reduction in the growth defect for the individual RRM deletions. It should be mentioned in the manuscript.

**Author’s comment:** Reduced protein level of ∆RRM2 in previous pap blot in Figure 3C, was because of less protein loaded as indicated by anti-PGK1 blot. We repeated the protein level western blot result and found protein level of all the mutants to be comparable with wild-type. The previous Figure 3C is replaced by a new western blot figure and the PAP bands have been quantitated in figure 3C for n=3 experiments and plotted in Figure 3D.

 5. In Figure 4A, the Edc3-mCherry panels do not align well with the GFP panels. This makes the results more difficult to interpret. The authors write that the western blot in Figure 4C shows comparable expression of WT and deltaRRM1+2 proteins. As the control bands (Pgk1) appear different in these two samples, quantification of this blot would make this statement more convincing.

**Author’s comment:** We have incorporated the changes suggested by the Reviewer in figure 4A. We have repeated western blot of Sbp1-GFP in wild-type and mutant background and replaced 4C with new image where total protein level in each lane is more comparable. In figure 4D, we have plotted graphical quantitation of bands obtained by anti-GFP antibody in each lane and normalized with anti-PGK1 antibody (protein loading control). RRM deletion mutants of Sbp1 seem to express comparable to wild-type.

# Peer review: Reviewer response for version 2

**DOI:** 10.21956/wellcomeopenres.17163.r37831

**Published:** 2020-03-03

**Authors:** David Tollervey

**Affiliations:** 1 Wellcome Trust Centre for Cell Biology, University of Edinburgh, Edinburgh, UK

The authors have suitably addressed my initial comments and I am happy to approve the revised manuscript.

Is the work clearly and accurately presented and does it cite the current literature?

Partly

If applicable, is the statistical analysis and its interpretation appropriate?

Not applicable

Are all the source data underlying the results available to ensure full reproducibility?

No source data required

Is the study design appropriate and is the work technically sound?

Partly

Are the conclusions drawn adequately supported by the results?

Partly

Are sufficient details of methods and analysis provided to allow replication by others?

Yes

Reviewer Expertise:

Gene expression, RNA processing, RNA-protein interactions, ribosome synthesis

I confirm that I have read this submission and believe that I have an appropriate level of expertise to confirm that it is of an acceptable scientific standard.

# Peer review: Reviewer response for version 2

**DOI:** 10.21956/wellcomeopenres.17163.r37998

**Published:** 2020-03-02

**Authors:** Jeffrey Wilusz

**Affiliations:** 1 Department of Microbiology, Immunology and Pathology, Colorado State University, Fort Collins, CO, USA

This is the first time that I’ve had the privilege to review this study involving mutational analyses of the Sbp1 translational regulator on cell growth and granule formation. Overall I feel that the study makes a measurable, albeit somewhat incremental, contribution to our understanding of the role of the RRM domains of this factor in yeast biology. I have several major issues as well as some minor points that should be addressed to make the study as compelling as possible.

 Major Points:

1.  Fig. 2C: The growth defect observed with the over expression of Sbp1 in this panel is not as dramatic as other panels – and thus the conclusion of no effect of the mutated phosphorylation sites is not as compelling as other studies in the figure. Thus I would recommend that the authors improve the quality of these data to better support their conclusion. Perhaps the original suggestion of including growth curves should be reconsidered.
2. Fig. 3C: The low expression of the delta RRM2 mutant does not allow clear conclusions to be made from this construct relative to the other constructs. This represents a fundamental flaw in the experiment that must be addressed.
3. Fig. 4C: The western blot clearly demonstrates that the RRM delta 1/2 protein is expressed much less than the WT protein in the experiment. This could be affecting the granule localization results and must to be addressed so that the conclusions drawn are fully convincing. As with the previous point, this represents a fundamental flaw in the experiment. 

Minor Points:

1.  It is important for authors to strive to polish the English language usage in their manuscripts to avoid unconscious bias by the reader that could affect the impact of their study. This is a potential issue with this manuscript in my opinion. There are numerous places in the manuscript, for example, where subject-verb agreement is lost which makes reading difficult. For example, the legend of Fig. 1. should be edited to read as follows (changes are in italics):
  “Multiple sequence alignment of Sbp1 reveals conservation of aromatic residue in the RGG motif and RNP sequence in the RRM domain. Blue indicate *s* negatively charged amino acids, purple indicate *s* positively charged amino acids, red indicate *s* hydrophobic amino acids, and green indicate *s* polar uncharged amino acid *s*.”
2. There are two other problems with Fig. 1 legend that need to be addressed. First, ‘genera’ not genuses, is the plural of genus. Second, the legend needs to indicate what the code of asterisks, colons and periods means to allow the reader to fully digest the figure.
3. Fig. 4B: The authors need to directly state what the red and green bars refer to in the legend to allow the graph to be readily interpreted.

Is the work clearly and accurately presented and does it cite the current literature?

Yes

If applicable, is the statistical analysis and its interpretation appropriate?

Yes

Are all the source data underlying the results available to ensure full reproducibility?

No source data required

Is the study design appropriate and is the work technically sound?

Partly

Are the conclusions drawn adequately supported by the results?

Partly

Are sufficient details of methods and analysis provided to allow replication by others?

Yes

Reviewer Expertise:

mRNA stability; RNA virus host interactions; RNA biology.

I confirm that I have read this submission and believe that I have an appropriate level of expertise to confirm that it is of an acceptable scientific standard, however I have significant reservations, as outlined above.

# Response

**Published:** 2021-08-03

**Authors:** Purusharth Rajyaguru

**Affiliations:** Indian Institute of Science, India

**Jeffrey Wilusz**

**Department of Microbiology, Immunology and Pathology, Colorado State University, Fort Collins, CO, USA**

 This is the first time that I’ve had the privilege to review this study involving mutational analyses of the Sbp1 translational regulator on cell growth and granule formation. Overall I feel that the study makes a measurable, albeit somewhat incremental, contribution to our understanding of the role of the RRM domains of this factor in yeast biology. I have several major issues as well as some minor points that should be addressed to make the study as compelling as possible.

**Author’s comment:** We thank the reviewer for all the comments.

 Major Points:

 1.Fig. 2C: The growth defect observed with the over expression of Sbp1 in this panel is not as dramatic as other panels – and thus the conclusion of no effect of the mutated phosphorylation sites is not as compelling as other studies in the figure. Thus I would recommend that the authors improve the quality of these data to better support their conclusion. Perhaps the original suggestion of including growth curves should be reconsidered.

**Author’s comment:** We have repeated growth assay of phosphomimetic mutants of Gal-SBP1 construct and replaced the older growth assay image with new one in Figure 2C. We agree with the limitation of spotting assay however within these limitations, we observe that the phosphomimetic mutants do not seem to affect the growth defect phenotype. Whether these mutations affect other phenotypes related to Sbp1 remains to be addressed.

 2. Fig. 3C: The low expression of the delta RRM2 mutant does not allow clear conclusions to be made from this construct relative to the other constructs. This represents a fundamental flaw in the experiment that must be addressed.

**Author’s comment:** We think reduced level of ∆RRM2 was because of less total protein as indicated by PGK1. We have looked at protein level of ∆RRM2 in our previous study as well (Bhatter *et al*., 2019) and did not find it to be compromised. However, we have repeated measuring levels of ∆RRM1+2 mutant in this study. Figure 3D shows the quantitation of PAP intensity as observed in Figure 3C after normalizing with Ponceau stained blot. Together, the western blot (Figure 3C) and its graphical quantitation (Figure 3D) suggests that the protein level of the RRM mutants of Sbp1 is not compromised.

 Fig. 4C: The western blot clearly demonstrates that the RRM delta 1/2 protein is expressed much less than the WT protein in the experiment. This could be affecting the granule localization results and must to be addressed so that the conclusions drawn are fully convincing. As with the previous point, this represents a fundamental flaw in the experiment.

**Author’s comment: **We have repeated protein level analysis of Sbp1-GFP ∆RRM1+2 and quantitated the band intensity with wild-type for n=3 experiments (Figure 4D). We do not observe protein level of ∆RRM1+2 mutant to be compromised as compared to wild-type.

 Minor Points:

 It is important for authors to strive to polish the English language usage in their manuscripts to avoid unconscious bias by the reader that could affect the impact of their study. This is a potential issue with this manuscript in my opinion. There are numerous places in the manuscript, for example, where subject-verb agreement is lost which makes reading difficult. For example, the legend of Fig. 1. should be edited to read as follows (changes are in italics):

 “Multiple sequence alignment of Sbp1 reveals conservation of aromatic residue in the RGG motif and RNP sequence in the RRM domain. Blue indicates negatively charged amino acids, purple indicates positively charged amino acids, red indicates hydrophobic amino acids, and green indicates polar uncharged amino acids.”

 There are two other problems with Fig. 1 legend that need to be addressed. First, ‘genera’ not genuses, is the plural of genus. Second, the legend needs to indicate what the code of asterisks, colons and periods means to allow the reader to fully digest the figure.

**Author’s comment: **We have done the changes in the text as suggested by the reviewer. We thank the reviewer for thorough analysis of the text.

 Fig. 4B: The authors need to directly state what the red and green bars refer to in the legend to allow the graph to be readily interpreted.

**Author’s comment:** We have incorporated the graph details in the legends.

# Peer review: Reviewer response for version 2

**DOI:** 10.21956/wellcomeopenres.17163.r37832

**Published:** 2020-02-24

**Authors:** Tracy Nissan

**Affiliations:** 1 University of Sussex, Brighton, UK

The revised manuscript examines the translational repressor Sbp1. It dissects the domains and motifs region repressors required to inhibit growth upon overexpression. It further examines its effect in different mutant backgrounds. The authors have satisfied my earlier comments. I have a few minor comments that can be addressed by alterations of the texts and figures.

1. Some statements remain stating that the results demonstrate translational repression, when the results are instead consistent with a translation repression effect. For example:
  - In the abstract: “We have further analyzed the importance of different domains and specific conserved residues of Sbp1 in translation repression activity”.
  - In the discussion: “This indicates that the site(s) required for Sbp1 interaction with mRNA and/or protein for translation repression function is not in the RNP motifs. 
  Without examining translation directly (for example in a cell extract), these are suggested by the results. The text should be re-written to make this clearer. 
2. The authors should indicate the FRG and RGG repeats in Figure 1 with boxes or shading. It is difficult to determine the frequency of these repeats in this figure.
3. Pat1 overexpression did not kill the cells in Figure 2D. This is interesting, since it has been reported by Pilkington and Parker, MCB 2008 ^1^ and Sopko *et al*., Genes Dev 2006 ^2^ among others. Since Pat1 overexpression does not cause cell death as previously reported, I am not sure it informative to keep this experiment in the manuscript. If they keep this data, they should indicate that Pat1 overexpression has been previously reported to be moderately lethal.
4. The “growth phenotype” and “overexpression phenotype” are used interchangeably in the manuscript. It would be clearer, if “growth phenotype” was changed to growth defect or similar language. 
5. The authors should indicate where the Sbp1 granules are in Figure 4A. It is not clear how many foci are present in the selected cells and where they overlap with Edc3 foci.
6. It would be helpful to add a small additional panel to Figure 2 to show the Sbp1 protein as a rectangle and the RRM and RNP regions indicated by shading and labelling. 
7. Supplementary tables are not accessible.
8. The methods section has some text that should have been removed in editing.

Is the work clearly and accurately presented and does it cite the current literature?

Yes

If applicable, is the statistical analysis and its interpretation appropriate?

Not applicable

Are all the source data underlying the results available to ensure full reproducibility?

Yes

Is the study design appropriate and is the work technically sound?

Yes

Are the conclusions drawn adequately supported by the results?

Partly

Are sufficient details of methods and analysis provided to allow replication by others?

Partly

Reviewer Expertise:

NA

I confirm that I have read this submission and believe that I have an appropriate level of expertise to confirm that it is of an acceptable scientific standard.

## References

[1] : Pat1 contains distinct functional domains that promote P-body assembly and activation of decapping.*Mol Cell Biol*.2008;28(4) : 10.1128/MCB.00936-071298-31210.1128/MCB.00936-0718086885PMC2258743

[2] : Mapping pathways and phenotypes by systematic gene overexpression.*Mol Cell*.2006;21(3) : 10.1016/j.molcel.2005.12.011319-3010.1016/j.molcel.2005.12.01116455487

# Response

**Published:** 2021-08-03

**Authors:** Purusharth Rajyaguru

**Affiliations:** Indian Institute of Science, India

**Tracy Nissan**

**University of Sussex, Brighton, UK**

 The revised manuscript examines the translational repressor Sbp1. It dissects the domains and motifs region repressors required to inhibit growth upon overexpression. It further examines its effect in different mutant backgrounds. The authors have satisfied my earlier comments. I have a few minor comments that can be addressed by alterations of the texts and figures.

 1. Some statements remain stating that the results demonstrate translational repression, when the results are instead consistent with a translation repression effect. For example:

 - In the abstract: “We have further analyzed the importance of different domains and specific conserved residues of Sbp1 in translation repression activity”.

 - In the discussion: “This indicates that the site(s) required for Sbp1 interaction with mRNA and/or protein for translation repression function is not in the RNP motifs. 

**Author’s comment:** We agree with the reviewers and have incorporated the changes in the manuscript.

 2. Without examining translation directly (for example in a cell extract), these are suggested by the results. The text should be re-written to make this clearer. The authors should indicate the FRG and RGG repeats in Figure 1 with boxes or shading. It is difficult to determine the frequency of these repeats in this figure.

**Author’s comment:** We have removed the translation repression activity term from the text. The FRG and RGG repeats are highlighted in figure 2A.

 3. Pat1 overexpression did not kill the cells in Figure 2D. This is interesting, since it has been reported by Pilkington and Parker, MCB 2008  and Sopko et al., Genes Dev 2006  among others. Since Pat1 overexpression does not cause cell death as previously reported, I am not sure it informative to keep this experiment in the manuscript. If they keep this data, they should indicate that Pat1 overexpression has been previously reported to be moderately lethal.

**Author’s comment:** We agree with the reviewer. According to the report of Pilkington and Parker, 2008, the fifth dilution of cell after spotting grows slow when Pat1 is over-expressed (Figure 6B). In our report in figure 2D, the fifth dilution of Gal-Pat1 transformed cell seems to grow slow as compared to empty-vector, as reported in literature.

 4. The “growth phenotype” and “overexpression phenotype” are used interchangeably in the manuscript. It would be clearer, if “growth phenotype” was changed to growth defect or similar language. 

**Author’s comment:** We have incorporated the changes in the manuscript text as suggested by the reviewer.

 5. The authors should indicate where the Sbp1 granules are in Figure 4A. It is not clear how many foci are present in the selected cells and where they overlap with Edc3 foci. It would be helpful to add a small additional panel to Figure 2 to show the Sbp1 protein as a rectangle and the RRM and RNP regions indicated by shading and labelling. 

**Author’s comment: **We have indicated Sbp1 granules with an arrow in Figure 4A. A cartoon with different domains of Sbp1 is incorporated in Figure 2A along with the sequence.

 6. Supplementary tables are not accessible.

**Author’s comment:** We have made it accessible

 7. The methods section has some text that should have been removed in editing.

**Author’s comment:** It has been removed

# Peer review: Reviewer response for version 1

**DOI:** 10.21956/wellcomeopenres.16019.r33743

**Published:** 2018-09-10

**Authors:** David Tollervey

**Affiliations:** 1 Wellcome Trust Centre for Cell Biology, University of Edinburgh, Edinburgh, UK

The authors report a mutational analysis of the RGG region of the protein Spb1. As an assay they use the dominant negative growth phenotype that was previously reported to be caused by over expression of Spb1. The chief problem in interpreting the results is that the basis of the growth phenotype remains unclear and none of the mutations tested clearly affected growth in the plate tests shown.

 Overall, while the results of the study are certainly worth recording as information for the field, this does not constitute a body of work that should be included in PubMed. I regret to write that I could not recommend acceptance for indexing without considerable additional work.

 Specific comments:

1. It might be expected that the aromatic residues within the RGG domain contribute to RNA interactions. However, the effects are not very marked at the level of growth. The enhanced growth for Mut1 and Mut2 relative to Spb1 is not readily visible in Figure 2C. Given that this alteration in growth rate is the only positive result reported in the MS, this conclusion should be more robustly supported.
  This should include growth curves with error bars indicating the reproducibility of the effects. This would also reveal whether the alteration affects maximal growth rate or some other feature of the growth curve.
  For plate tests it would also be better to inoculate with cultures grown to defined OD, rather than patches of cells grown overnight, which might be different between strains.
2. The authors test phosphomimetic mutants and conclude that these do not affect growth. Is there reason to believe that these sites are ever phosphorylated?

Is the work clearly and accurately presented and does it cite the current literature?

Partly

If applicable, is the statistical analysis and its interpretation appropriate?

Not applicable

Are all the source data underlying the results available to ensure full reproducibility?

No source data required

Is the study design appropriate and is the work technically sound?

Partly

Are the conclusions drawn adequately supported by the results?

Partly

Are sufficient details of methods and analysis provided to allow replication by others?

Yes

Reviewer Expertise:

Gene expression, RNA processing, RNA-protein interactions, ribosome synthesis

I confirm that I have read this submission and believe that I have an appropriate level of expertise to confirm that it is of an acceptable scientific standard, however I have significant reservations, as outlined above.

# Response

**Published:** 2020-01-17

**Authors:** Purusharth Rajyaguru

**Affiliations:** Indian Institute of Science, India

**Author’s response**

We thank the reviewer for the comments on this manuscript.

Specific comments:

1. It might be expected that the aromatic residues within the RGG domain contribute to RNA interactions. However, the effects are not very marked at the level of growth. The enhanced growth for Mut1 and Mut2 relative to Spb1 is not readily visible in Figure 2C. Given that this alteration in growth rate is the only positive result reported in the MS, this conclusion should be more robustly supported. This should include growth curves with error bars indicating the reproducibility of the effects. This would also reveal whether the alteration affects maximal growth rate or some other feature of the growth curve. For plate tests it would also be better to inoculate with cultures grown to defined OD, rather than patches of cells grown overnight, which might be different between strains.

**Author’s response**

We agree with the reviewer and have changed the conclusion after repeating growth assay for Mut 1 and 2. The growth alteration for Mut1 and Mut2 are not very notable.

To bolster the impact of the work we have now incorporated two results (Figure 3 & 4) that demonstrate the positive role of RRM domains in Sbp1 function, which has not been reported earlier. We provide evidence that the RRM domains are required for Sbp1 overexpression mediated growth defect and for localization of Sbp1 to granules. We think these results significantly elevate the importance of the manuscript.

2. The authors test phosphomimetic mutants and conclude that these do not affect growth. Is there reason to believe that these sites are ever phosphorylated?

**Author’s response**

We have incorporated the reference that reported Sbp1 phosphorylation at the specific residue that we mutated in the manuscript.

# Peer review: Reviewer response for version 1

**DOI:** 10.21956/wellcomeopenres.16019.r33744

**Published:** 2018-08-23

**Authors:** Tracy Nissan

**Affiliations:** 1 University of Sussex, Brighton, UK

This manuscript addresses the ability of the translational repressors Sbp1, Dhh1 and Pat1 to inhibit growth and therefore likely repress translation. The contribution of this work is to demonstrate that mutated residues in Sbp1’s RGG motif in contrast to other RGG proteins are apparently not important for its function. In addition, the manuscript addresses the importance of Scd6 for growth inhibition of Sbp1 and whether the absence of Sbp1 would affect the growth inhibition caused by overexpression of Dhh1 or Pat1. This work provides a further step for understanding the residues important for Sbp1 translational repression as assessed by growth inhibition as well as providing more insight into the genetic relations between translational repressors. Suggestions for addressing my reservations are indicated below:

 1. Since the ability to repress translation was not directly tested, the authors should alter the statement at the end of the Conclusion on p1: “Interestingly Scd6 does not affect ability of Sbp1 to repress translation, which in turn does not affect Dhh1 and Pat1.” to indicate that it likely does not affect the ability to repress translation or similar statement.

 2. It would be helpful to indicate under what conditions is Sbp1 phosphorylated in previous studies (p3) to assist in interpretation of the results of the phosphomimetic experiment (Fig 3B). 

 3. I cannot see the difference between the growth inhibition of Sbp1 with the phosphomimetic mutants in Figure 3B.  It would be helpful to indicate where the growth was marginally less inhibited. In addition, a statement of how many times the experiment was performed should be indicated.

 4. A reference should be added for the plasmid pIR6 in the materials section.  In addition the regions cloned into that plasmid from the SBP1 gene should be indicated.

 5. There is a mistake on p4, where Ded1 is discussed instead of Dhh1.

Is the work clearly and accurately presented and does it cite the current literature?

Yes

If applicable, is the statistical analysis and its interpretation appropriate?

Not applicable

Are all the source data underlying the results available to ensure full reproducibility?

Yes

Is the study design appropriate and is the work technically sound?

Yes

Are the conclusions drawn adequately supported by the results?

Partly

Are sufficient details of methods and analysis provided to allow replication by others?

Partly

Reviewer Expertise:

NA

I confirm that I have read this submission and believe that I have an appropriate level of expertise to confirm that it is of an acceptable scientific standard, however I have significant reservations, as outlined above.

# Response

**Published:** 2020-01-17

**Authors:** Purusharth Rajyaguru

**Affiliations:** Indian Institute of Science, India

**Author’s response**

The authors thank the reviewer for comments on this manuscript.

We would like to bring to notice of the reviewer that we have now incorporated two results (Figure 3 & 4) that demonstrate the role of RRM domains in Sbp1 function. This has not been reported earlier. We provide evidence that the RRM domains are required for Sbp1 overexpression mediated growth defect and for localization of Sbp1 to granules. We think these results significantly elevate the importance of the manuscript.

The manuscript has been revised and rewritten to interpret the new data and to address reviewer's concerns.

*Suggestions for addressing my reservations are indicated below:*

1. Since the ability to repress translation was not directly tested, the authors should alter the statement atthe end of the Conclusion on p1: “Interestingly Scd6 does not affect ability of Sbp1 to repress translation, which in turn does not affect Dhh1 and Pat1.” to indicate that it likely does not affect the ability to repress translation or similar statement.

**Author’s response**

We have done the changes in the text.

2. It would be helpful to indicate under what conditions is Sbp1 phosphorylated in previous studies (p3) to assist in interpretation of the results of the phosphomimetic experiment (Fig 3B).

**Author’s response**

We have included the information on the text. (line 133).

3. I cannot see the difference between the growth inhibition of Sbp1 with the phosphomimetic mutants in Figure 3B.  It would be helpful to indicate where the growth was marginally less inhibited. In addition, a statement of how many times the experiment was performed should be indicated.

**Author’s response**

We have not observed any impact of the phosphomimetic mutants on the Sbp1 overexpression mediated growth defect phenotype and concluded accordingly.

4. A reference should be added for the plasmid pIR6 in the materials section.  In addition the regions cloned into that plasmid from the SBP1 gene should be indicated.

**Author’s response**

We have incorporated the details in the text (line 278)

5. There is a mistake on p4, where Ded1 is discussed instead of Dhh1.

**Author’s response**

We have incorporated the changes.
